# SSBD: a database of quantitative data of spatiotemporal dynamics of biological phenomena

**DOI:** 10.1093/bioinformatics/btw417

**Published:** 2016-07-13

**Authors:** Yukako Tohsato, Kenneth H. L. Ho, Koji Kyoda, Shuichi Onami

**Affiliations:** Laboratory for Developmental Dynamics, RIKEN Quantitative Biology Center, Kobe 650-0047, Japan

## Abstract

**Motivation**: Rapid advances in live-cell imaging analysis and mathematical modeling have produced a large amount of quantitative data on spatiotemporal dynamics of biological objects ranging from molecules to organisms. There is now a crucial need to bring these large amounts of quantitative biological dynamics data together centrally in a coherent and systematic manner. This will facilitate the reuse of this data for further analysis.

**Results**: We have developed the Systems Science of Biological Dynamics database (SSBD) to store and share quantitative biological dynamics data. SSBD currently provides 311 sets of quantitative data for single molecules, nuclei and whole organisms in a wide variety of model organisms from *Escherichia coli* to *Mus musculus*. The data are provided in Biological Dynamics Markup Language format and also through a REST API. In addition, SSBD provides 188 sets of time-lapse microscopy images from which the quantitative data were obtained and software tools for data visualization and analysis.

**Availability and Implementation**: SSBD is accessible at http://ssbd.qbic.riken.jp.

**Contact:** sonami@riken.jp

## 1 Introduction

One of the leading challenges of systems biology is to understand the nature of the dynamical behaviors of biological phenomena. Recent progress in live-cell imaging techniques has produced a large amount of microscopy images showing the spatiotemporal dynamics of biological objects such as single molecules, nuclei, cells and organisms (Keller, 2013). Computational image analysis techniques can quantitatively extract numerical data from these microscopy images (Peng, 2008; Sommer and Gerlich, 2013). These quantitative biological dynamics data can then be analyzed further to provide crucial insight into the nature of dynamical behaviors of biological phenomena.

Many sets of quantitative biological dynamics data have been obtained from various kinds of microscopy images. For example, Bao *et al.* (2006) extracted quantitative data of nuclear division dynamics in green fluorescent protein-labeled embryos using confocal microscopy with the aim of deciphering the cell lineage in *Caenorhabditis elegans*. Kyoda *et al.* (2013) extracted quantitative data of nuclear division dynamics in *C.elegans* embryos under a wide variety of gene perturbations from differential interference contrast microscopy images to understand molecular mechanisms in early embryogenesis. Similarly, quantitative data of nuclear division dynamics in embryos were obtained for *Drosophila melanogaster* (Keller *et al.*, 2010) and *Danio rerio* (Keller *et al.*, 2008) using digital scanned laser light-sheet microscopy. These quantitative data allowed a comprehensive analysis of cell division patterns during embryogenesis. Cronin *et al.* (2005) extracted quantitative data of behavioral dynamics of adult *C.elegans* to understand how genes influence behavior and locomotion.

Quantitative biological dynamics data can be reused for further analysis of dynamical behaviors of biological phenomena; however, only a few existing datasets have been reused even though most are publicly available. For example, quantitative data of nuclear division dynamics in *D.rerio* embryos produced by Keller *et al.* (2008) were reused to analyze spatial organization of cells with the use of newly developed information metrics (Hoh *et al.*, 2013), and quantitative data of nuclear division dynamics in *C.elegans* embryos were reused to evaluate image-processing methods for nuclear detection (Azuma and Onami, 2013; Santella *et al.*, 2010). There are two reasons for the low rate of data reuse. One is that the data are usually dispersed on individual websites across the Internet, so researchers often find it difficult to know what kinds of data are available. The other reason is that incompatible data formats create a barrier for other researchers to reuse the data because studying each individual format takes additional time and effort.

Recent progress in mathematical modeling techniques has provided an opportunity to perform mechanobiological simulations, which also generate quantitative data. Quantitative data of spatiotemporal dynamics of single molecules in an *Escherichia coli* cell were generated to elucidate the mechanism of pole-to-pole oscillations of target proteins (Arjunan and Tomita, 2010). Data of microtubule-dependent pronuclear migration in early *C.elegans* embryos were generated to reveal the mechanism of the nuclear centering process (Kimura and Onami, 2005). In such simulation studies, data from computer simulations are often compared with *in vivo* dynamic patterns from biological experiments to evaluate the model validity. Once a plausible model is created, the model can predict dynamical behaviors over a range of parameter values. The deviation in predictions can be reused leading to further experiments and refinement of the models (Mogilner *et al.*, 2006). However, such kinds of quantitative data from computer simulation are neither stored nor shared.

There is now a crucial need to bring large amounts of quantitative data of spatiotemporal dynamics together centrally in a coherent and systematic manner to facilitate the reuse of the data for further analysis. Several research groups are developing central databases that store and share data in the field of bioimage informatics (Lemberger, 2015; Swedlow *et al.*, 2009). The Cell Image Library (Orloff *et al.*, 2013) and the Image Data Repository (http://idr-demo.openmicroscopy.org) store and share microscopy images with meta-information. However, these databases mainly focus on microscopy images and do not store and share quantitative data extracted from images. The Biostudies database (McEntyre *et al.*, 2015) stores and shares meta-information of biological studies, and it provides links to data sources for a wide range of biological datasets. Moreover, there are also several databases that provide quantitative biological dynamics data in the field of computer simulation. However, these databases were very specific for storing data from molecular dynamics simulations (Meyer *et al.*, 2010; Van der Kamp *et al.*, 2010) and biochemical kinetic simulations (Karr *et al.*, 2014). Thus far there is no central database that stores and shares quantitative data of spatiotemporal dynamics obtained from bioimage informatics techniques or mechanobiological modeling techniques.

In this paper, we present the Systems Science of Biological Dynamics database (SSBD; http://ssbd.qbic.riken.jp) for storing and sharing quantitative biological dynamics data. This database is developed and maintained with support from Japan’s National Bioscience Database Center (NBDC; http://biosciencedbc.jp/en/) as a part of the Life Science Database Integration Project in Japan (http://biosciencedbc.jp/en/about-us/projects-and-activities). NBDC is part of the Japan Science and Technology Agency (JST). The Ministry of Education, Culture, Sports, Science and Technology of Japan (MEXT) requests all researchers in the life sciences in Japan to provide their data to support the project. SSBD was developed to sustainably store and share quantitative biological dynamics data that are created by the Japanese science community and beyond. It provides users with central access to quantitative data, and the microscopy images from which the quantitative data were obtained. It also provides additional software tools for data visualization and analysis.

## 2 Methods

### 2.1 Concept behind SSBD

SSBD is designed to store and share quantitative data of spatiotemporal dynamics of biological objects ranging from single molecules to organisms in a coherent and systematic manner. The data stored in SSBD can be accessed in two systematic ways. The first way is to download a dataset with the use of a unified format for representing quantitative biological dynamics data called Biological Dynamics Markup Language (BDML; Kyoda *et al.*, 2015). BDML is the only open format that supports a wide variety of types of quantitative biological dynamics data. The second way is through the use of a REST API (Representational State Transfer Application Programming Interface; Fielding and Taylor, 2002). In addition, SSBD stores and shares microscopy images from which the quantitative data were obtained, and offline software tools to access BDML files for data visualization and analysis. Furthermore, the SSBD website allows users to visualize quantitative data and microscopy images directly in a web browser. SSBD is a unique database for data-driven biology in that it allows users to access large sets of diverse quantitative data together with microscopy images and software tools.

### 2.2 Data collection and annotation

SSBD currently only provides quantitative biological dynamics data published in peer-reviewed journal papers. This policy is to ensure that the data have been peer-reviewed and are of acceptable quality. Because the data are often described in their original data formats stored on authors’ websites, we currently convert each of them into BDML format (Kyoda *et al.*, 2015) and then make that data available in SSBD with the authors’ permissions. As mentioned above, SSBD was originally developed to store and share all quantitative biological dynamics data produced publicly by the Japanese science community; however, we also encourage international research groups to use SSBD to store and share their quantitative data. Therefore, SSBD is open to requests from all research scientists who would like to provide their data (http://ssbd.qbic.riken.jp/contact/).

When using quantitative data in SSBD, users should be able to refer back to the original paper. Therefore, each dataset is annotated with a PubMed identifier. Names and contact information of the corresponding authors for the article are also stored as meta-information within SSBD.

License information is annotated based on the permission granted by the corresponding authors of the article and the owner of the dataset. The individual license information allows both data providers and data users to easily share their data. SSBD recommends data to be distributed under the terms of the Creative Commons License (http://creativecommons.org/licenses/). This will encourage and facilitate further sharing and reuse of quantitative data.

### 2.3 Database design

SSBD stores three types of resources: quantitative data with meta-information, microscopy images from which the quantitative data were obtained, and software tools for data visualization and analysis.

**Fig. 1.**
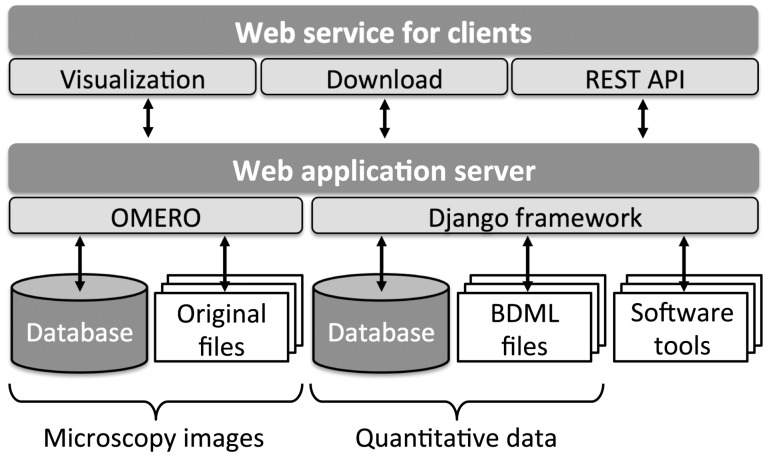
Quantitative data in BDML format and microscopy images from which quantitative data were obtained are visualized via a web browser. Users can obtain the data and images together with BDML-compatible software tools

In SSBD, quantitative data are stored in two different representations. Each dataset is stored as a BDML formatted file and a set of tables within a relational database (Fig. 1). The BDML file allows users to download the complete dataset directly, whereas the relational database allows users to search and find similarities across the entire database. Relational tables also allow users to have direct access to a specific part of the quantitative data through the REST API without downloading the whole dataset (see ‘Web services’ section). We chose to use a relational database rather than a native XML database because relational databases give better response times during searches and have much better tools and support for software and web service development (Li *et al.*, 2010).

SSBD also stores the microscopy images from which the quantitative data were obtained. Internal to SSBD, the images are handled by the Open Microscopy Environment Remote Objects (OMERO) software platform (Fig. 1; Allan *et al.*, 2012). OMERO supports over 130 image file formats including all major microscope formats; therefore, most microscopy images can be managed without the concern of incompatible image file formats. Meta-information embedded in the images, such as pixel size, time interval and configuration of microscope system, is automatically stored in a relational database and can be accessed by the users.

SSBD is also a repository for sharing BDML-compatible software tools for data visualization and analysis. All software tools are stored in the file system of SSBD and are downloadable at http://ssbd.qbic.riken.jp/software/ without any registration requirement.

## 3 Implementation

### 3.1 SSBD implementation

SSBD is implemented on a Red Hat Enterprise Linux server release 6.6 with Apache HTTP server 2.2.15. The current release of SSBD is built on a Django 1.5.2 web application framework running Python 2.6.6. Quantitative data are stored in a PostgreSQL 8.4.20 relational database. Microscopy images are managed by OMERO 5.0.4 running on Django 1.6 and Java OpenJDK 1.7.0_45.

The SSBD REST API was implemented using the Tastypie 0.10 framework for Django. A reference implementation using Python and Java applications to access the REST API can be found at https://github.com/openssbd/.

The browser-based 4D viewer for quantitative data is implemented using JavaScript based on the three.js r66 framework, jQuery 1.10.2 and jQuery-UI 1.10.4 library. The three.js framework is based on Web Graphics Library (WebGL) whereas jQuery utilizes Asynchronous JavaScript and XML (AJAX) technology. All modern browsers support both WebGL and AJAX without additional plugin or software installation.

### 3.2 Software implementation

SSBD currently provides software tools named BDML4DViewer and Phenochar for visualization and analysis, respectively, of quantitative data in BDML format (see ‘Software tools’ section for details). BDML4DViewer is implemented as a plugin of ImageJ (Schneider *et al.*, 2012) using the Java programming language. Java Architecture for XML Binding (JAXB) and Java Binding for the OpenGL (JOGL) APIs are required for installing this plugin. Source codes and the executable JAR file for this plugin are available online at http://ssbd.qbic.riken.jp/BDML4DViewer/. Phenochar is implemented in the C programming language. CodeSynthesis XSD is required to compile this tool. Source codes of this tool are available online at http://ssbd.qbic.riken.jp/phenochar/.

SSBD also provides a plugin of ImageJ named SSBD-OMERO.insight-ij to access the microscopy images stored in SSBD. This plugin was created by modifying the login functions of the original OMERO.insight-ij software, which was released by the OME consortium. The executable JAR file for this plugin is available online at http://ssbd.qbic.riken.jp/SSBD-OMERO.insight/.

OpenSSBD is the open-source version of SSBD for managing quantitative biological dynamics data. The current release of OpenSSBD is implemented in Python 2.7.6 using a Django 1.6.1 and PostgreSQL 9.3.10 relational database engine on the Ubuntu 14.04 operating system. All source codes and additional REST API for importing data from BDML files into the relational database are available at https://github.com/openssbd/. A Docker container for OpenSSBD is also available at https://hub.docker.com/r/openssbd/public/.

## 4 Current resources

### 4.1 Quantitative data

**Fig. 2.**
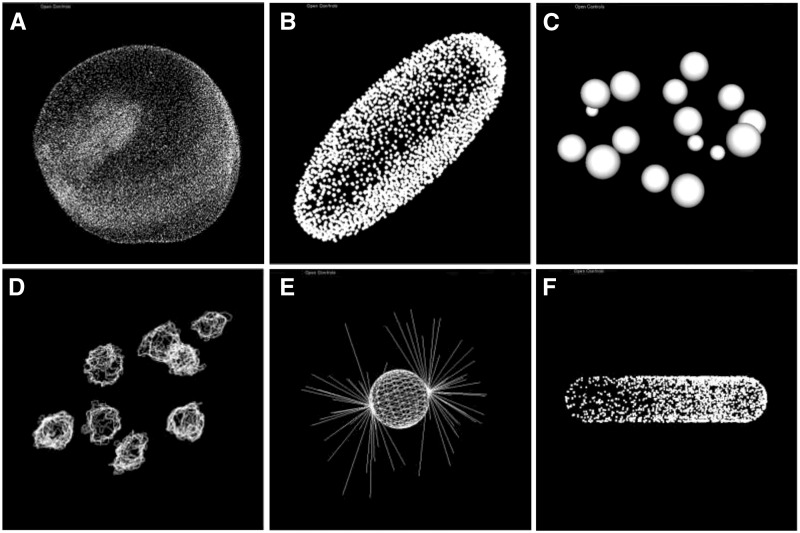
Visualization of quantitative data extracted from experimental measurements or predicted from computer simulations. Nuclear division dynamics data extracted from microscopy images of *Danio rerio* (Keller *et al.*, 2008) (**A**), *Drosophila melanogaster* (Keller *et al.*, 2010) (**B**) and *Caenorhabditis elegans* (Bao *et al.*, 2006; Kyoda *et al.*, 2013) (**C, D**) embryos. Data predicted from computer simulations of microtubule-dependent pronuclear migration in *C.elegans* embryos (Kimura and Onami, 2005) (**E**) and single molecule dynamics in an *Escherichia coli* cell (Arjunan and Tomita, 2010) (**F**)

SSBD currently provides 311 sets of quantitative data of biological dynamics (Table 1; Fig. 2). Various types of quantitative data of biological objects ranging from single molecules to organisms are available for a wide variety of model organisms. The data extracted from microscopy images include

**Table 1.** List of available resources in SSBD

Reference	Organism	Dynamics	#sets of BDML	#sets of images
Experimental measurements				
Bashar *et al*. (2012)	*Mus musculus*	Nucleus	1	1
Keller *et al*. (2008)	*Danio rerio*	Nucleus	7	0
Keller *et al*. (2010)	*Drosophila melanogaster*	Nucleus	2	0
Bao *et al*. (2006)	*Caenorhabditis elegans*	Nucleus	2	0
Kyoda *et al*. (2013)	*Caenorhabditis elegans*	Nucleus	186	186
Cronin *et al*. (2005)	*Caenorhabditis elegans*	Behavior	11	0
Komatsuzaki *et al*. (2015)	*Dictyostelium discoideum*	Single molecule	1	1
Simulation results				
Kimura and Onami (2005)	*Caenorhabditis elegans*	Pronucleus and microtubule	100	0
Arjunan and Tomita (2010)	*Escherichia coli*	Single molecule	1	0

- one set of nuclear division dynamics data of wild-type embryos from the 17- to 33-cell stage in *Mus musculus* (Bashar *et al.*, 2012),
- seven sets of embryogenesis data from about 1.5 h postfertilization (h.p.f.) up to 30 h.p.f. in *D.rerio*, including those from wild-type and *one-eye pinhead* mutant embryos (Keller *et al.*, 2008; Fig. 2A),
- two sets of embryogenesis data from 2 h.p.f. to 11.5 h.p.f. in *D.melanogaster* (Keller *et al.*, 2010; Fig. 2B),
- one set of nuclear division dynamics data of wild-type embryo from 4- to 350-cell stage in *C.elegans* (Bao *et al.*, 2006; Fig. 2C),
- fifty sets of nuclear division dynamics data of wild-type *C.elegans* embryos from one- to eight-cell stages and 136 sets of nuclear division dynamics of RNAi-treated *C. elegans* embryos corresponding to 72 essential embryonic genes on chromosome III (Kyoda *et al.*, 2013; Fig. 2D),
- eleven sets of behavioral data of *C.elegans* adults (Cronin *et al.*, 2005) and
- one set of single molecule dynamics data of G-protein-coupled receptors in a *Dictyostelium discoideum* cell (Komatsuzaki *et al.*, 2015).

In addition to quantitative data extracted from microscopy images, SSBD also provides simulation results, including

- one hundred sets of microtubule-dependent pronuclear migration data in early *C.elegans* embryos (Kimura and Onami, 2005; Fig. 2E) and
- one set of single molecule dynamics data of Min proteins in an *E.coli* cell (Arjunan and Tomita, 2010; Fig. 2F).

### 4.2 Microscopy images

SSBD provides 188 sets of microscopy images from which quantitative data were obtained (Table 1). These sets include

- one set of three-dimensional (3D) time-lapse confocal microscopy images recording early development in a *M.musculus* wild-type embryo at 10 min intervals for about 17 h (Bashar *et al.*, 2012),
- one hundred eighty-six sets of 3D time-lapse differential interference contrast microscopy images recording early development of 50 wild-type and 136 RNAi-treated *C.elegans* embryos for 72 essential embryonic genes on chromosome III at 40 s intervals for 2 h (Kyoda *et al.*, 2013) and
- one set of time-lapse internal reflection fluorescence microscopy images recording single molecules in a *D.discoideum* wild-type cell at 0.033 s intervals for about 1 min (Komatsuzaki *et al.*, 2015).

In total, the datasets comprise approximately 2.2 million microscopy images in z-stacks and time-lapse series.

### 4.3 Software tools

SSBD provides software tools for data visualization and analysis of quantitative data that use the BDML format. It also provides software to directly read microscopy images from SSBD. An open-source version of SSBD is also made available. This software is currently licensed under GNU GPLv3 to ensure that users are free to use, modify, enhance and share their contribution with the community. A complete list of this software is available at http://ssbd.qbic.riken.jp/software/.

#### 4.3.1 BDML4DViewer

BDML4DViewer is a software tool implemented as a plugin of ImageJ for interactively visualizing quantitative data in BDML format (Fig. 2). Time series of 3D spatial data represented as predefined geometric entities such as points, lines, spheres, faces and their combinations can be viewed using a mouse and keyboard.

#### 4.3.2 Phenochar

Phenochar is a standalone software tool for extracting various kinds of phenotypic characters from quantitative data in BDML format: e.g. rate of increase in the number of biological objects such as nuclei over time and changes in spatial displacement of objects over time. It can be used to compare data from different laboratories even when different microscopy equipment was used.

#### 4.3.3 SSBD-OMERO.insight-ij

SSBD-OMERO.insight-ij is a software tool implemented as a plugin of ImageJ for accessing the microscopy images stored in SSBD. This plugin enables the user to directly read and analyze the microscopy images.

#### 4.3.4 OpenSSBD

OpenSSBD is the open-source version of SSBD for managing quantitative data. It enables each individual scientist or research group to set up their own database on their own server to independently store and share their quantitative data. It provides the essential functions of SSBD, e.g. a simple keyword search function, REST API direct access to quantitative data, and a simple browser-based viewer for visualization of quantitative data.

## 5 Web services

### 5.1 Keyword search

**Fig. 3.**
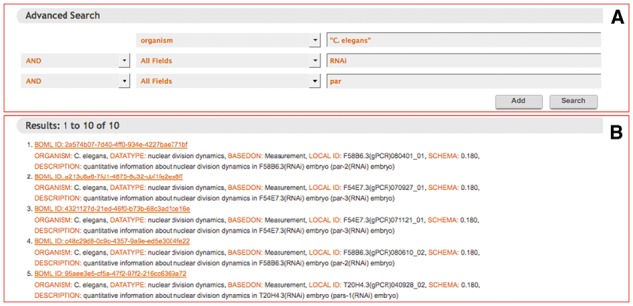
Screenshot of a keyword search result. Panel (**A**) shows a completed input form for a keyword search. Keywords can be combined by the logical operators AND, OR, or NOT. Panel (**B**) shows a search result

**Fig. 4.**
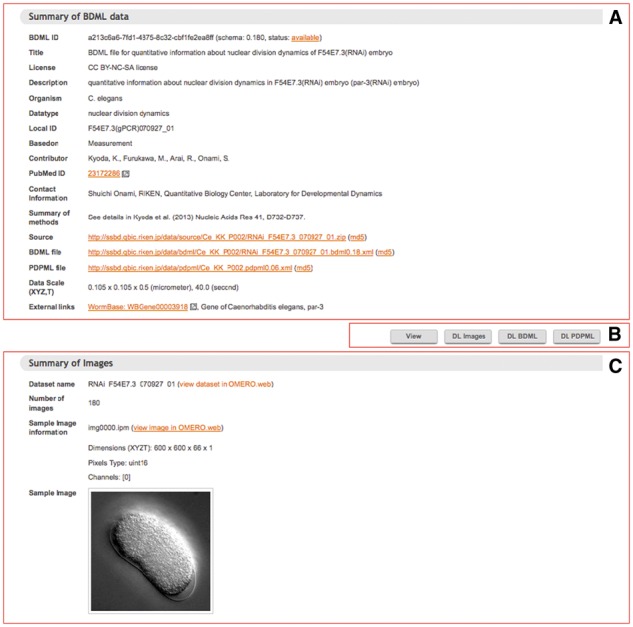
Screenshot of a summary page of quantitative data. Panel (**A**) shows meta-information about the quantitative data. Panel (**B**) shows the four buttons that allow the user to choose to view the quantitative data, or download the microscopy images, or BDML or PDPML (Procedure for Data Processing Markup Language; Kyoda *et al.*, 2015) formatted files for the quantitative data. PDPML files describe the procedures used to obtain quantitative data, either from image analysis techniques or from computer simulations. BDML and PDPML files use human readable filenames. For example, in ‘RNAi_F54E7.3_070927_01.bdml0.18.xml’, a BDML file name for quantitative data of nuclear division dynamics of *C.elegans* embryos, ‘RNAi_F54E7.3’ indicates that the open reading frame of the gene *F54E7.3* has been inactivated by RNAi, and ‘bdml0.18’ indicates that the file uses BDML version 0.18 format. Panel (**C**) appears when the images are available in SSBD. This panel displays the meta-information about the microscopy images with a link to the page of the OMERO platform

Users can enter a keyword search that looks for matching text in the title, description, contact information, schema version and other meta-information of all the quantitative data stored in the database (Fig. 3). They can search by combining the logical operators AND, OR and NOT. The search result returns links to summary pages for individual datasets together with some meta-information. A summary page allows the users to visualize and download not only the quantitative data but also the corresponding microscopy images when those images are available (Fig. 4). This page can be directly accessed using bdmlID, which is a unique identifier of the BDML file: for an example, see http://ssbd.qbic.riken.jp/search/df2a9568-9c33-4b48-b138-46548bccff6d/.

### 5.2 Data visualization on web browser

**Fig. 5.**
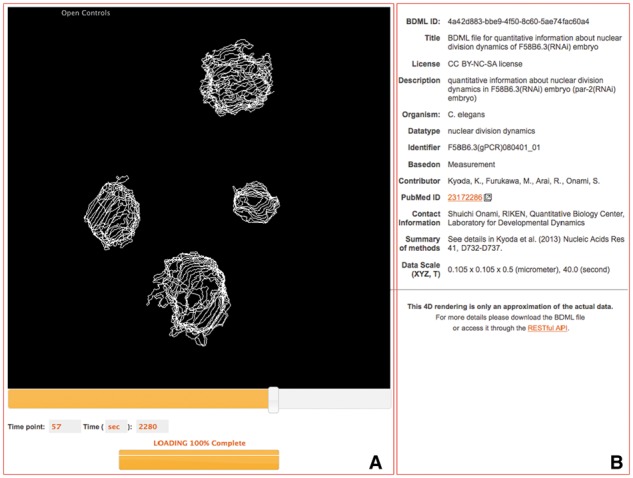
Screenshot of an interactive 4D view of quantitative data. Panel (**A**) shows a 4D visualization of the quantitative data. Users can change the angle and scale of objects in the viewer by using the mouse, and the time point by using the scroll bar and keyboard. The viewer supports data loading in the background, the status of which is indicated by a progress bar at the bottom of the screen. Panel (**B**) shows meta-information about the quantitative data

Visualization of quantitative data is important to understand elements of biological dynamics such as position and movement. A browser-based four-dimensional (4D) viewer was developed to visualize on-demand the quantitative data in SSBD (Fig. 5). It allows users to visualize time series of 3D spatial data without first downloading the dataset. Users can explore SSBD datasets online before choosing the relevant data to download for further analysis. Quantitative data is visualized as 4D models on a web browser. Users can change view angles and time points with mouse operations. Data is updated in the background without reloading the entire web page.

### 5.3 REST API

**Fig. 6.**
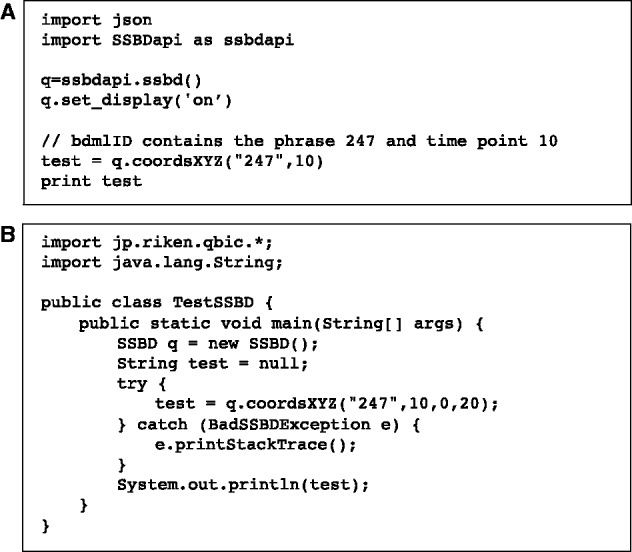
Example data request using a REST API in Python (**A**) and Java (**B**). More details at https://github.com/openssbd

Users can directly access quantitative data stored in SSBD by means of an API based on the REST pattern (Fielding and Taylor, 2002). REST uses the same Hypertext Transfer Protocol (HTTP) that a web browser uses to request and receive data via Uniform Resource Locators (URLs). SSBD REST API is a simple web-based service interface allowing any programming language (e.g. Python, Java) to have direct access to the SSBD data. Output supports both JavaScript Object Notation (JSON) and XML formats. Figure 6 shows example code of data requests in Python and Java; details of the examples can be found at https://github/openssbd. Detailed documentation on the SSBD REST API is provided at http://ssbd.qbic.riken.jp/restfulapi/. The SSBD REST API can be used to access a portion of data by setting limits to a prescribed time point.

### 5.4 Linked meta-information

SSBD provides Resource Description Framework (RDF; http://www.w3.org/TR/rdf11-concepts/) formatted meta-information of the quantitative data and their corresponding microscopy images at RIKEN Meta Database (http://metadb.riken.jp/metadb/db/SSBD). It allows data on SSBD to be searched by other databases. RDF is the current trend in linking different databases together (Jupp *et al.*, 2014; Katayama *et al.*, 2013). The SSBD-RDF data consists of 18 319 triples (data entities of subject–predicate–object form). The SPARQL query language (SPARQL; http://www.w3.org/TR/rdf-sparql-query/) can be used on the website to query the SSBD-RDF data. Detailed documentation about the semantic relationships of SSBD-RDF data and examples of SPARQL queries are provided at http://ssbd.qbic.riken.jp/rdf/.

SSBD also provides links to three external databases: Ensembl (Yates *et al.*, 2015; release 76 version 2018.8) for genomic information, WormBase (Yook *et al.*, 2012; version WS246) for genetic information in *C.elegans* and PubMed (http://www.pubmed.gov) for scientific literature. We will add external links to various external databases for genome and genetic information according to the needs when storing new quantitative data obtained from gene knockout or knockdown experiments.

## 6 Applications of SSBD

**Fig. 7.**
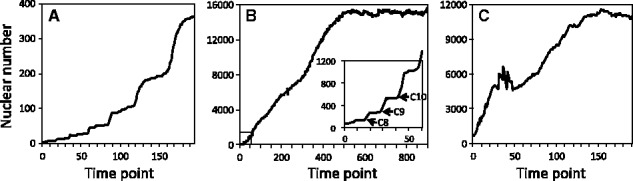
Time-dependent proliferation pattern in embryogenesis of *Caenorhabditis elegans* (**A**), *Danio rerio* (**B**) and *Drosophila melanogaster* (**C**). Inset: synchronization of nuclear division at the 9th cycle (C9) and the start of loss of synchronization thereafter (C10) during early embryogenesis. Nuclear number was calculated by applying the software tool Phenochar to the BDML files of *C.elegans* (Bao *et al.*, 2006), *D.rerio* (Keller *et al.*, 2008) and *D.melanogaster* (Keller *et al.*, 2010) in SSBD

To demonstrate how quantitative data stored in SSBD can be reused to understand biological processes, we used the database to analyze time-dependent proliferation patterns during embryogenesis in *D.rerio* and *D.melanogaster* (Fig. 7).

Previous studies in *C.elegans* showed that time-dependent proliferation pattern analysis provides insights into the mechanisms of development (Bao *et al.*, 2008; Deppe *et al.*, 1978; Sulston *et al.*, 1983). In *C.elegans*, a stepwise increase in nuclear number throughout embryogenesis was observed; this increase was shown to originate from synchronous cell divisions of the descendants of the ‘AB’ founder cell (Sulston *et al.*, 1983; Fig. 7A). To conduct time-dependent proliferation pattern analysis of *D.rerio* and *D.melanogaster*, we calculated nuclear number throughout embryogenesis from the quantitative data of *D.rerio* (Keller *et al.*, 2008) and *D.melanogaster* (Keller *et al.*, 2010); such calculations can be made by applying Phenochar (see ‘Software tools’ section) to the BDML files stored in SSBD or using the SSBD REST API.

In *D.rerio*, we found a precise stepwise pattern in the early stage of embryogenesis (Fig. 7B). The stepwise pattern was gradually broken at around the 10th zygotic cell cycle, and the stepwise pattern started to transition to a linear pattern. This result suggests that cell divisions are synchronous in the early stage and become asynchronous around the 10th zygotic cell cycle in the whole embryo. This result is consistent with the observations reported previously for time-lapse recordings of part of a *D.rerio* embryo: cell cycle lengthening was first observed in most cells at the 10th zygotic cell cycle and varied in extent (Kane and Kimmel, 1993). It will be intriguing to uncover the molecular mechanisms that switch cell divisions from synchronous to asynchronous in a whole embryo.

In *D.melanogaster*, a rapid stepwise increase in nuclear number was also observed until the 50th time point, which corresponds to 4.5 h.p.f. (Fig. 7C). This increase is consistent with well-known phenomena called mitotic waves (Foe and Alberts, 1983). We found a temporary decrease in the nuclear number at the 50th time point (4.5 h.p.f.). Such a decrease might be caused by cell death, cell fusion, or errors in nuclear detection. It is likely that the observed decrease was caused at least in part by errors in nuclear detection because the time point corresponds to the onset of mesoderm internalization. Mesoderm internalization affects the performance of the image-processing method for nuclear detection (Keller *et al.*, 2010). This result suggests that even a feature in the time-dependent proliferation pattern caused by errors in nuclear detection can be informative. Such a feature may reflect a change in the mode of biological processes. Further analysis will be needed to clarify the relative contribution of cell death, cell fusion and errors in nuclear detection to the observed decrease. To this end, the original image data that were used to produce the quantitative data are desired. SSBD can provide a platform to publish such original image data.

These results indicate that time-dependent proliferation analysis can provide insights into synchrony and asynchrony of cell or nuclear divisions, and other biological processes such as cell death, cell fusion and cell internalization during embryogenesis. This analysis demonstrates how quantitative data stored in SSBD can be reused to understand biological processes.

## 7 Discussion

SSBD is a database for storing and sharing quantitative biological dynamics data for biological objects of various scales, ranging from single molecules to organisms. Over 310 sets of quantitative data of biological dynamics can be reused in BDML formatted (Kyoda *et al.*, 2015) files and through the SSBD REST API. As an example of the reuse of data in SSBD, we examined time-dependent proliferation patterns during embryogenesis in several model organisms. The data can also be reused for comparative analysis. For example, we can directly compare the data from different laboratories when the data pertains to the same biological phenomena in the same model organism. Moreover, we can compare data obtained from related or different species to reveal similar and different features (Zhao *et al.*, 2008). Furthermore, we might combine different types of data such as cell morphology and protein activity data (Tsukada *et al.*, 2008) to explore the relationship between the underlying biophysical and biochemical changes.

We store simulation results within SSBD, enabling direct comparison with quantitative data extracted from biological experiments. Comparison between the extracted data under gene perturbation and the simulation results with perturbed parameters may provide a mechanistic insight into gene function (Kimura and Onami, 2007). However, it is difficult to determine what type of simulation results should be stored and shared because an infinite number of simulation results can be generated by varying each parameter in a mathematical model. It may be appropriate to store and share the computer programs and mathematical models that produce the results in the future. Meanwhile we plan to store simulation results that are expensive to produce, for example, simulations that take months to run on a supercomputer. These results cannot be easily reproduced in researchers’ individual laboratories.

Data quality control is a major problem in most biological databases, and SSBD encounters the same problem. However, SSBD is different from other databases such as genome and gene expression databases because it also stores the original microscopy images from which the quantitative biological dynamics data were obtained. By visually checking the quantitative data with the original microscopy image data, one can directly evaluate the quality of the quantitative data. Online and offline visualization tools are available in SSBD, enabling easy evaluation of the data quality.

Microscopy images stored in SSBD can provide new opportunities for computational biologists, especially those in the field of bioimage informatics. It is possible to develop new methods for extracting quantitative data of new biological objects from existing microscopy images because these images often contain information that the original study did not focus on or utilize. Other possibilities include the development of new or improved methods for extracting quantitative data of the same biological objects examined in the original study. The performance of these new methods can then be evaluated by comparing their output with the data stored in SSBD. The Broad Bioimage Benchmark Collection (BBBC; Ljosa *et al.*, 2012) is a database for storing annotated microscopy image sets for testing image-processing methods. Each image set is provided with the corresponding quantitative data such as contours of biological objects extracted by image analysis. BBBC aims to improve image-processing algorithms for biological microscopy, whereas SSBD aims to provide quantitative biological resources for bioinformaticians and computational biologists to further advance biological research. It might be possible for the BBBC and SSBD projects to collaborate and share a similar software platform to enhance image-processing algorithms as well as to provide quantitative data resources for biologists in the future.

We distributed an open-source version of SSBD, OpenSSBD, as a software platform for managing quantitative biological dynamics data. It has the essential functions of SSBD with a browser-based simple interactive 4D viewer and the same REST API for accessing data. Several groups have developed open-source platforms that can manage numerical information of biological dynamics. The OMERO platform was developed primarily to manage microscopy images but it can also manage quantitative data pertaining to regions of interest (ROIs) based on the 2D geometric models in OME (Allan *et al.*, 2012). However, a limitation of OMERO is that it cannot use 3D geometric models such as spheres and faces in BDML. The openBIS platform was developed to manage biological research data, including microscopy images, and numerical information from high-content screening (Bauch *et al.*, 2011). However, openBIS is not suitable for managing spatiotemporal information about biological dynamics. All these other platforms do not provide an interactive 4D viewer for quantitative data because they are not specialized for storing and sharing quantitative data. Therefore, OpenSSBD is a unique software platform for managing quantitative biological dynamics data.

OpenSSBD enables research groups to develop their own databases to store and share their quantitative data. The distribution of OpenSSBD could result in acceleration of data sharing with the ‘data bazaar’ approach (Poldrack and Gorgolewski, 2014), although it could also lead to data scattering across the Internet. To avoid such data scattering, a community-based effort for managing data integration is indispensable; for instance, a central database could be used to store and share at least the meta-information about all the quantitative data, including their download sites. If all quantitative data are managed under the control of the community, data storage, processing and download services can be dispersed throughout the databases managed by research groups in the community. SSBD can be viewed as a ‘data factory’ approach (Poldrack and Gorgolewski, 2014). Currently, it uses a central database to store and share all quantitative biological dynamics data created by the Japanese scientific community. However, an international community-based effort is required for efficient, effective and sustainable data sharing in the era of open science.

To make the operation of SSBD sustainable for the future, we plan to develop a registration system enabling researchers and users to register and then upload their original data in BDML format. In addition, the current policy of SSBD is to store only data published in peer-reviewed journals to ensure that the data have been reviewed. This policy provides adequate confidence in the accuracy of the data stored in SSBD. However, we are likely to change this policy in the future to accept quantitative data before publication. This will allow authors to reference the data within their unpublished paper and will allow editors and reviewers to access those data before publication. SSBD will be required to introduce the concept of accession number, which provides a unique alphanumeric identifier for each dataset. One possible solution is to use the bdmlID (Kyoda *et al.*, 2015). SSBD will also need a new attribute to denote the publication status of the data to ensure that users can have confidence in the accuracy of the data. SSBD will play a larger role as a public repository for quantitative biological dynamics data in the near future.

## 8 Conclusion

SSBD is a unique database that enables scientists in a wide variety of fields to reuse the large amount of quantitative biological dynamics data obtained from biological experiments and computer simulations. SSBD will support, promote and contribute to advances in systems biology and various interdisciplinary research fields, and facilitate data-driven biology.

## References

[btw417-B1] Allan C. (2012) OMERO: flexible, model-driven data management for experimental biology. Nat. Methods, 9, 245–253. 22373911 10.1038/nmeth.1896 PMC3437820

[btw417-B2] Arjunan S.N. Tomita M. (2010) A new multicompartmental reaction-diffusion modeling method links transient membrane attachment of *E. coli* MinE to E-ring formation. Syst. Synth. Biol., 4, 35–53. 20012222 10.1007/s11693-009-9047-2 PMC2816228

[btw417-B3] Azuma Y. Onami S. (2013) Evaluation of the effectiveness of simple nuclei-segmentation method on *Caenorhabditis elegans* embryogenesis images. BMC Bioinf., 14, 295. 10.1186/1471-2105-14-295 PMC4077036 24090283

[btw417-B4] Bao Z. (2006) Automated cell lineage tracing in *Caenorhabditis elegans.* Proc. Natl. Acad. Sci. U. S. A., 103, 2707–2712. 16477039 10.1073/pnas.0511111103 PMC1413828

[btw417-B5] Bao Z. (2008) Control of cell cycle timing during *C. elegans* embryogenesis. Dev. Biol., 318, 65–72. 18430415 10.1016/j.ydbio.2008.02.054 PMC2442716

[btw417-B6] Bauch A. (2011) openBIS: a flexible framework for managing and analyzing complex data in biology research. BMC Bioinf., 12, 468. 10.1186/1471-2105-12-468 PMC3275639 22151573

[btw417-B7] Bashar M.K. (2012) Automatic extraction of nuclei centroids of mouse embryonic cells from fluorescence microscopy images. PLoS One, 7, e355550. 10.1371/journal.pone.0035550 PMC3348125 22590505

[btw417-B8] Cronin C.J. (2005) An automated system for measuring parameters of nematode sinusoidal movement. BMC Genet., 6, 5. 15698479 10.1186/1471-2156-6-5 PMC549551

[btw417-B9] Deppe U. (1978) Cell lineages of the embryo of the nematode *Caenorhabditis elegans.* Proc. Natl. Acad. Sci. U. S. A., 75, 376–380. 272653 10.1073/pnas.75.1.376 PMC411251

[btw417-B10] Fielding R.T. Taylor R.N. (2002) Principled design of the modern Web architecture. ACM Trans. Internet Technol., 2, 115–150.

[btw417-B11] Foe V.E. Alberts B.M. (1983) Studies of nuclear and cytoplasmic behaviour during the five mitotic cycles that precede gastrulation in *Drosophila* embryogenesis. J. Cell Sci., 61, 31–70. 6411748 10.1242/jcs.61.1.31

[btw417-B12] Hoh J.H. (2013) Spatial information dynamics during early zebrafish development. Dev. Biol., 377, 126–137. 23438813 10.1016/j.ydbio.2013.02.005

[btw417-B13] Jupp S. (2014) The EBI RDF platform: linked open data for the life sciences. Bioinformatics, 30, 1338–1339. 24413672 10.1093/bioinformatics/btt765 PMC3998127

[btw417-B14] Kane D.A. Kimmel C.B. (1993) The zebrafish midblastula transition. Development, 119, 447–456. 8287796 10.1242/dev.119.2.447

[btw417-B15] Karr J.R. (2014) WholeCellSimDB: a hybrid relational/HDF database for whole-cell model predictions. Database, 2014. 10.1093/database/bau095 PMC4165886 25231498

[btw417-B16] Katayama T. (2013) The 3rd DBCLS BioHackathon: improving life science data integration with Semantic Web technologies. J. Biomed. Semantics, 4, 6. 23398680 10.1186/2041-1480-4-6 PMC3598643

[btw417-B17] Keller P.J. (2008) Reconstruction of zebrafish early embryonic development by scanned light sheet microscopy. Science, 322, 1065–1069. 18845710 10.1126/science.1162493

[btw417-B18] Keller P.J. (2010) Fast, high-contrast imaging of animal development with scanned light sheet-based structured-illumination microscopy. Nat. Methods, 7, 637–642. 20601950 10.1038/nmeth.1476 PMC4418465

[btw417-B19] Keller P.J. (2013) Imaging morphogenesis: technological advances and biological insights. Science, 340, 1234168. 23744952 10.1126/science.1234168

[btw417-B20] Kimura A. Onami S. (2005) Computer simulations and image processing reveal length-dependent pulling force as the primary mechanism for *C. elegans* male pronuclear migration. Dev. Cell, 8, 765–775. 15866166 10.1016/j.devcel.2005.03.007

[btw417-B21] Kimura A. Onami S. (2007) Local cortical pulling force repression switches centrosomal centration and posterior displacement in *C. elegans.* J. Cell Biol., 179, 1347–1354. 18158330 10.1083/jcb.200706005 PMC2373484

[btw417-B22] Komatsuzaki A. (2015) Compact halo-ligand-conjugated quantum dots for multicolored single-molecule imaging of overcrowding GPCR proteins on cell membranes. Small, 11, 1396–1401. 25504902 10.1002/smll.201402508

[btw417-B23] Kyoda K. (2013) WDDD: Worm Developmental Dynamics Database. Nucleic Acids Res., 41, D732–D737. 23172286 10.1093/nar/gks1107 PMC3531189

[btw417-B24] Kyoda K. (2015) Biological Dynamics Markup Language (BDML): an open format for representing quantitative biological dynamics data. Bioinformatics, 31, 1044–1052. 25414366 10.1093/bioinformatics/btu767 PMC4382901

[btw417-B25] Lemberger T. (2015) Image data in need of a home. Mol. Syst. Biol., 11, 853. 26700853 10.15252/msb.20156719 PMC4704486

[btw417-B26] Li C. (2010) BioModels database: an enhanced, curated and annotated resource for published quantitative kinetic models. BMC Syst. Biol., 4, 92. 20587024 10.1186/1752-0509-4-92 PMC2909940

[btw417-B27] Ljosa V. (2012) Annotated high-throughput microscopy image sets for validation. Nat. Methods, 9, 637. 22743765 10.1038/nmeth.2083 PMC3627348

[btw417-B28] McEntyre J. (2015) The BioStudies database. Mol. Syst. Biol., 11, 847. 26700850 10.15252/msb.20156658 PMC4704487

[btw417-B29] Meyer T. (2010) MoDEL (Molecular Dynamics Extended Library): a database of atomistic molecular dynamics trajectories. Structure, 18, 1399–1409. 21070939 10.1016/j.str.2010.07.013

[btw417-B30] Mogilner A. (2006) Quantitative modeling in cell biology: what is it good for? Dev. Cell, 11, 279–287. 16950120 10.1016/j.devcel.2006.08.004

[btw417-B31] Orloff D.N. (2013) The cell: an image library-CCDB: a curated repository of microscopy data. Nucleic Acids Res., 41, D1241–D1250. 23203874 10.1093/nar/gks1257 PMC3531121

[btw417-B32] Peng H. (2008) Bioimage informatics: a new area of engineering biology. Bioinformatics, 24, 1827–1836. 18603566 10.1093/bioinformatics/btn346 PMC2519164

[btw417-B33] Poldrack R.A. Gorgolewski K.J. (2014) Making big data open: data sharing in neuroimaging. Nat. Neurosci., 17, 1510–1517. 25349916 10.1038/nn.3818

[btw417-B34] Santella A. (2010) A hybrid blob-slice model for accurate and efficient detection of fluorescence labeled nuclei in 3D. BMC Bioinformatics, 11, 580. 21114815 10.1186/1471-2105-11-580 PMC3008706

[btw417-B35] Schneider C.A. (2012) NIH Image to ImageJ: 25 years of image analysis. Nat. Methods, 9, 671–675. 22930834 10.1038/nmeth.2089 PMC5554542

[btw417-B36] Sommer C. Gerlich D.W. (2013) Machine learning in cell biology - teaching computers to recognize phenotypes. J. Cell Sci., 126, 5529–5539. 24259662 10.1242/jcs.123604

[btw417-B37] Sulston J.E. (1983) The embryonic cell lineage of the nematode *Caenorhabditis elegans.* Dev. Biol., 100, 64–119. 6684600 10.1016/0012-1606(83)90201-4

[btw417-B38] Swedlow J.R. (2009) Bioimage informatics for experimental biology. Ann. Rev. Biophys., 38, 327–346. 19416072 10.1146/annurev.biophys.050708.133641 PMC3522875

[btw417-B39] Tsukada Y. (2008) Quantification of local morphodynamics and local GTPase activity by edge evolution tracking. PLoS Comput. Biol., 4, e1000223. 19008941 10.1371/journal.pcbi.1000223 PMC2573959

[btw417-B40] Van der Kamp M.W. (2010) Dynameomics: a comprehensive database of protein dynamics. Structure, 18, 423–435. 20399180 10.1016/j.str.2010.01.012 PMC2892689

[btw417-B41] Yates A. (2015) The Ensembl REST API: Ensembl data for any language. Bioinformatics, 31, 143–145. 25236461 10.1093/bioinformatics/btu613 PMC4271150

[btw417-B42] Yook K. (2012) WormBase: more genomes, more data, new website. Nucleic Acids Res., 40, D735–D741. 22067452 10.1093/nar/gkr954 PMC3245152

[btw417-B43] Zhao Z. (2008) Comparative analysis of embryonic cell lineage between *Caenorhabditis briggsae* and *Caenorhabditis elegans.* Dev. Biol., 314, 93–99. 18164284 10.1016/j.ydbio.2007.11.015 PMC2696483

